# Dysphagia following transcatheter mitral valve-in-ring replacement complicated by neo-left ventricular outflow tract obstruction: a case report

**DOI:** 10.1093/ehjcr/ytaf081

**Published:** 2025-02-14

**Authors:** Stephanie Sargent, Eddy Xiong, Katherine Lau, Owen Christopher Raffel, Kim Greaves

**Affiliations:** Cardiology Department, Sunshine Coast University Hospital, 6 Doherty Street, Birtinya, 4575 Queensland, Australia; School of Medicine, Griffith University, 6 Doherty Street, Birtinya, 4575 Queensland, Australia; Cardiology Department, The Prince Charles Hospital, 627 Rode Road, Chermside, 4032 Queensland, Australia; Cardiology Department, Sunshine Coast University Hospital, 6 Doherty Street, Birtinya, 4575 Queensland, Australia; School of Medicine, Griffith University, 6 Doherty Street, Birtinya, 4575 Queensland, Australia; Cardiology Department, The Prince Charles Hospital, 627 Rode Road, Chermside, 4032 Queensland, Australia; School of Clinical Medicine, The University of Queensland, 288 Herston Road, Herston, 4006 Queensland, Australia; Cardiology Department, The Prince Charles Hospital, 627 Rode Road, Chermside, 4032 Queensland, Australia; School of Clinical Medicine, The University of Queensland, 288 Herston Road, Herston, 4006 Queensland, Australia; School of Mechanical, Medical & Process Engineering, Faculty of Science & Engineering, Queensland University of Technology, 2 George Street, Brisbane City, 4000 Queensland, Australia; Cardiology Department, Sunshine Coast University Hospital, 6 Doherty Street, Birtinya, 4575 Queensland, Australia; School of Medicine, Griffith University, 6 Doherty Street, Birtinya, 4575 Queensland, Australia

**Keywords:** Echocardiography, Case report, Dysphagia, Transcatheter mitral valve replacement, Trans-septal mitral-valve-in-ring, Intravascular haemolysis, Neo-LVOT obstruction

## Abstract

**Background:**

Neo-left ventricular outflow tract (LVOT) obstruction is a dreaded complication following transcatheter mitral valve replacement (TMVR). Dynamic LVOT obstruction has been reported to cause mechanical intravascular haemolysis due to red cell fragmentation. Intravascular haemolysis can result in a rare but well-described phenomenon in which patients experience dysphagia due to oesophageal spasm. This phenomenon is classically associated with paroxysmal nocturnal haemoglobinuria and has never been reported following TMVR.

**Case summary:**

A 77-year-old female presented 8 days following TMVR with presyncope, dyspnoea, and severe dysphagia. Transthoracic echocardiography revealed neo-LVOT obstruction with trivial paravalvular mitral regurgitation. Doppler echocardiography revealed dynamic late-peaking LVOT obstruction with a peak gradient of 71 mmHg with Valsalva manoeuvre. Laboratory investigations demonstrated intravascular haemolytic anaemia. A barium swallow was performed revealing severe diffuse oesophageal spasm. Alternative causes of dysphagia were excluded, and a causal link between intravascular haemolysis and dysphagia was identified. Successful management of dynamic neo-LVOT obstruction, haemolytic anaemia, and dysphagia was achieved with beta blocker therapy and volume resuscitation. Serial echocardiography and barium swallow performed prior to discharge demonstrated resolution of LVOT obstruction and marked reduction in oesophageal spasm.

**Discussion:**

To our knowledge, this is the first case report of TMVR complicated by reversible neo-LVOT obstruction with only trivial paravalvular regurgitation causing intravascular haemolysis and subsequent dysphagia. The management of dysphagia secondary to intravascular haemolysis due to neo-LVOT obstruction is challenging. This is because of the complex haemodynamic interplay between reduced oral intake, a high output state with anaemia, increased ventricular contractility, tachycardia, and worsening dynamic obstruction, all part of a vicious cycle (*Summary figure*).

**Learning points**

- Intravascular haemolysis secondary to neo-left ventricular outflow tract (LVOT) obstruction can lead to dysphagia due to nitric oxide depletion and subsequent smooth muscle spasm and dysmotility.
- The management of dysphagia in this setting is challenging due to the complex haemodynamic interplay of volume depletion, increased ventricular contractility, tachycardia, and worsening neo-LVOT obstruction.
- Successful management of dynamic neo-LVOT obstruction, haemolytic anaemia, and dysphagia was achieved with beta blocker therapy and volume resuscitation.

## Introduction

Transcatheter mitral valve replacement (TMVR) is an emerging minimally invasive strategy for the management of severe symptomatic mitral regurgitation in patients who are considered high surgical risk. Due to the complexity of the mitral valve apparatus and its proximity to the left ventricular outflow tract (LVOT), neo-LVOT obstruction is a dreaded complication of TMVR. Whilst pre-procedural planning with computerized tomography (CT) is an essential step in determining patient suitability for TMVR, there is a dynamic component to the development of LVOT obstruction which needs consideration.^1^

Dynamic LVOT obstruction is a well-known sequelae of hypertrophic cardiomyopathy, and can produce intravascular haemolysis due to increased velocity of blood flow across the LVOT resulting in red blood cell destruction.^2^ Intravascular haemolysis can lead to dysphagia due to oesophageal spasm as a result of nitric oxide sequestration by free haemoglobin. This is a well reported phenomenon in patients with paroxysmal nocturnal haemoglobinuria occurring in up to 51% of patients.^3^ The link between intravascular haemolysis and dysphagia in patients with cardiac conditions has only been reported in two published case reports, in patients with left ventricular assist devices.^4,5^

Intravascular haemolysis following TMVR has been reported in the setting of neo-LVOT obstruction and concurrent significant paravalvular leak. Whilst haemolytic anaemia is a known complication following TMVR, it is usually in the setting of significant paravalvular regurgitation. This is the first case report, to our knowledge to describe dynamic neo-LVOT obstruction following TMVR without significant paravalvular regurgitation resulting in intravascular haemolysis and dysphagia.

## Summary figure

**Figure:**
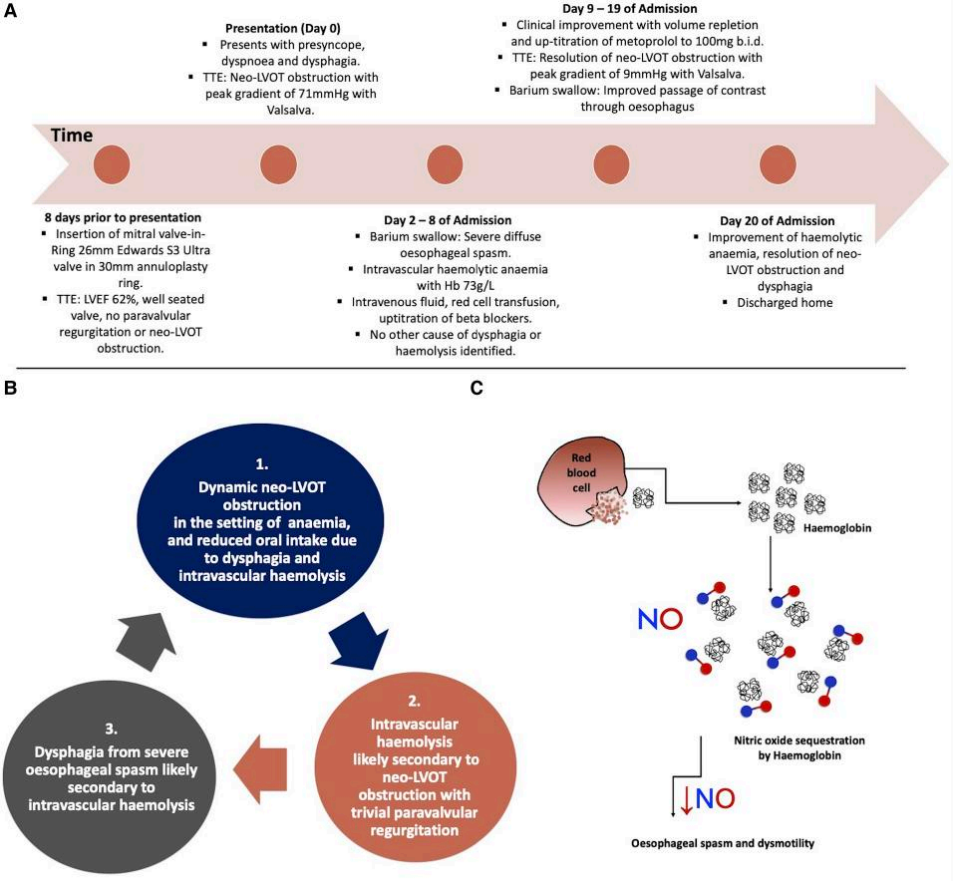


## Case presentation

**Figure 1:**
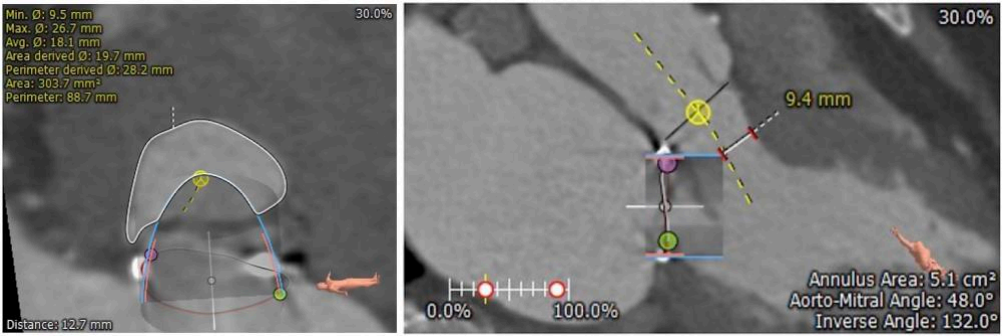
Pre-procedural computed tomography evaluation of the risk of LVOT obstruction. Predicted Neo-LVOT area and aorto-mitral angle based on virtual deployment of a 26 mm Edwards Sapien S3 valve are shown. A neo-LVOT area of 303.7 mm^2^ (>200 mm^2^) and an aorto-mitral angle of 132° (>90°) indicate a low risk of LVOT obstruction.

A 77-year-old female who had previously undergone mitral valve repair with a 30 mm annuloplasty ring and P2 neochordae for mitral valve prolapse presented with severe symptomatic mitral regurgitation. Her comorbidities included rheumatoid arthritis on sulfasalazine 500 mg twice daily, chronic kidney disease, and hypertension on perindopril 2.5 mg once daily. She was deemed too high risk for re-do sternotomy with a EuroSCORE II of 11% and subsequently underwent a trans-septal transcatheter mitral valve-in-ring (TMVinR) with implantation of a 26 mm Edwards S3 Ultra transcatheter heart valve. The preoperative CT deemed her anatomy appropriate for TMVinR with a CT neo-LVOT area of 303.7 mm^2^ and short anterior mitral valve leaflet (*Figure 1*). She was discharged Day 1 following a successful and uncomplicated procedure on metoprolol 25 mg twice daily, frusemide 40 mg daily, aspirin 100 mg daily, and warfarin. Her transthoracic echocardiogram prior to discharge demonstrated normal left ventricular function with an ejection fraction of 62%, a well-seated valve, trivial transvalvular regurgitation without paravalvular regurgitation or neo-LVOT obstruction.

**Figure 2:**
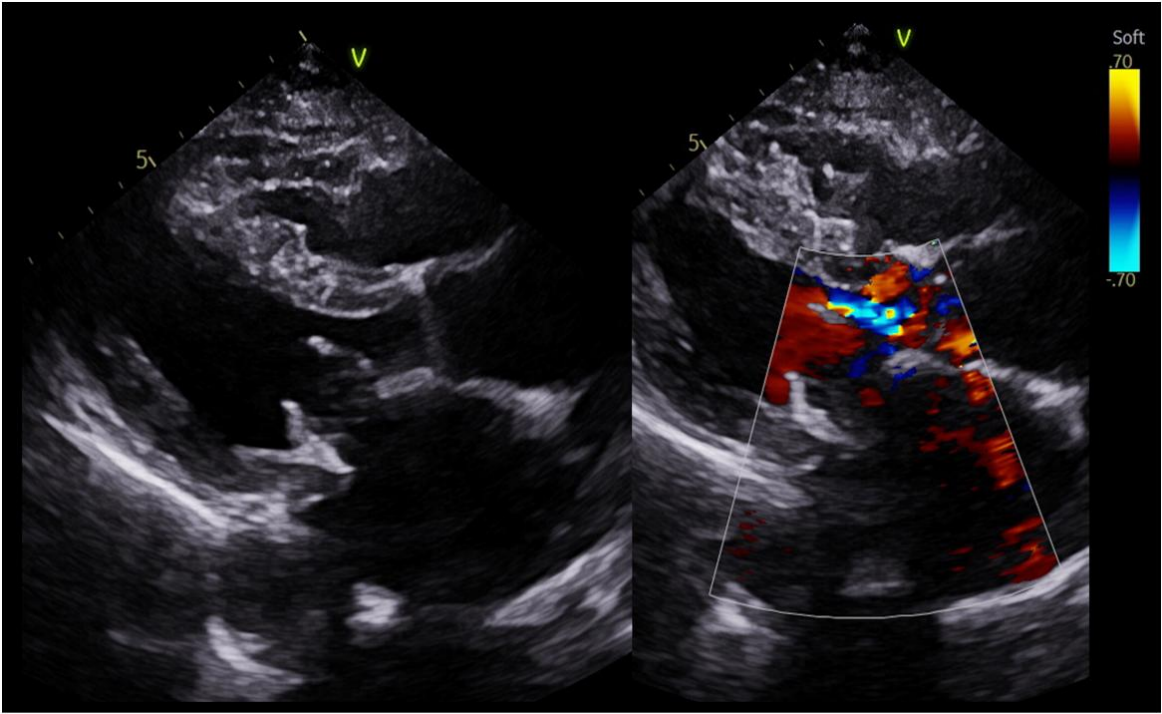
Echocardiography. Parasternal long axis demonstrating crowding of the LVOT with chordal SAM secondary to native anterior mitral valve leaflet. Flow acceleration within the LVOT secondary to Edwards S3 Ultra within 30 mm Physio ring (26 mm).

**Figure 3:**
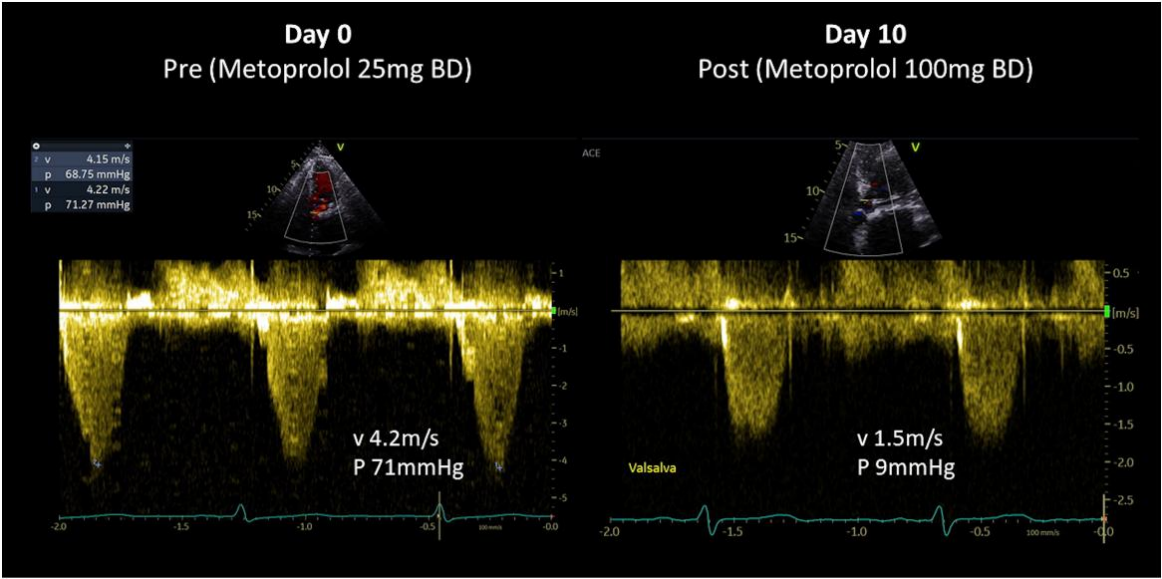
Continuous wave Doppler through the LVOT. Late peaking systolic signal suggestive of dynamic LVOT obstruction secondary to native mitral leaflets with a peak gradient 71 mmHg with Valsalva manoeuvre. Repeat echocardiography 10 days later showing an improvement in gradients across the LVOT with a peak gradient of 9 mmHg with Valsalva manoeuvre.

Eight days following her discharge, she presented to the emergency department with dyspnoea, presyncope, and dysphagia. Physical examination revealed a blood pressure of 90/60 mmHg, heart rate of 96 b.p.m., intravascular depletion, and a late peaking ejection systolic murmur. Laboratory parameters showed a haemoglobin (Hb) of 81 g/L (reference range: 115–165 g/L), lactate dehydrogenase (LDH) 2711 U/L (reference range: 120–250 U/L), and bilirubin 29 μmol/L (reference range: <20 μmol/L). Her echocardiogram upon re-presentation demonstrated LVOT obstruction secondary to native anterior mitral leaflet displacement with a peak gradient of 71 mmHg with Valsalva and trivial paravalvular regurgitation (*Figures 2* and *3* and Supplementary material online, *Video S1*). Metoprolol was up-titrated to 50 mg twice daily, perindopril was withheld, and intravenous fluid therapy was administered.

Two days into her admission, she developed an acute severe intravascular haemolytic anaemia with a Hb nadir of 73 g/L, LDH 4022 U/L, bilirubin 47 μmol/L, reticulocytes 277 × 10^9^/L (reference range: 20–120 × 10^9^/L), haptoglobin 0.01 g/L (reference range: 0.3–2.0 g/L), and schistocytes on blood film. Her dysphagia progressed and was unable to tolerate any oral intake. She required multiple blood transfusions, and intravenous fluid therapy was continued.

Haematology consultation was sought. Sulfasalazine was temporarily withheld as a potential drug-induced cause for haemolysis, however following haematology and immunology consultation, this was deemed an unlikely cause and sulfasalazine was re-commenced. After alternative causes for haemolysis were excluded including a CT neck to pelvis to investigate for possible malignancy, intravascular haemolysis was presumed secondary to cardiac pathology.

After seeking gastroenterology and speech pathology reviews, her dysphagia was investigated with a barium swallow which revealed diffuse oesophageal spasm (see Supplementary material online, *Video S2A*). She underwent an endoscopy which was normal. After excluding other causes for her dysphagia, a causal link between intravascular haemolysis and dysphagia was identified.

During her inpatient stay, beta blockers were up-titrated to maximally tolerated doses (metoprolol 100 mg twice daily). She had symptomatic improvement with resolution of dyspnoea, presyncope, and dysphagia. Her repeat echocardiogram prior to discharge demonstrated resolution of LVOT obstruction with a peak gradient with Valsalva of 9 mmHg (*Figure 3*, Supplementary material online, *Video S3*). A follow-up barium swallow revealed marked improvement in oesophageal spasm (see Supplementary material online, *Video S2B*). Her laboratory parameters improved with Hb 95 g/L, LDH 898 U/L, and bilirubin 7 μmol/L at discharge. Perindopril and frusemide were discontinued, and she was discharged on warfarin for three months post-procedure, metoprolol 100 mg twice daily, and sulfasalazine 500 mg twice daily.

## Discussion

Mitral regurgitation is the most common valvular pathology worldwide, affecting an estimated 2% of the global population and ∼10% of patients over the age of 75 years.^6^ An estimated 50% of patients with mitral regurgitation are not suitable operative candidates which has led to the development of minimally invasive interventions, such as the TMVR.^7^

The risk of neo-LVOT obstruction following TMVR is ∼7%–9% and up to 12% for trans-septal TMVinR procedures. It is a feared complication which carries mortality risk.^1^ In contemporary clinical trials, ∼50% of patients have a threatened projected neo-LVOT on pre-procedural CT screening and therefore pre-procedural CT is considered a crucial step in appropriate procedural planning and success.^1,8^ Left ventricular outflow tract obstruction is a dynamic process which results in reduced cardiac output and occurs due to a complex interplay between anatomical factors and physiological status. Whilst, pre-procedural CT can accurately model the projected post-procedural neo-LVOT area and risk for LVOT obstruction, certain haemodynamic factors including heart rate, left ventricular contractility, and volume status which are less predictable with significant variability can influence the neo-LVOT and these factors need to be considered.^9^

Clinically relevant haemolysis with symptomatic anaemia is rare following TMVR with an estimated incidence of ∼3%.^10,11^ Intravascular haemolysis following TMVR is often a result of paravalvular regurgitation; however, it has been described once in the literature in the setting of neo-LVOT obstruction with concurrent significant paravalvular regurgitation.^9^ Haemolysis secondary to LVOT obstruction has also been reported in patients with hypertrophic obstructive cardiomyopathy due to the turbulence of high-velocity blood flow and subsequent shearing stress on red blood cells.^12^

Dysphagia secondary to intravascular haemolysis is well reported in patients with paroxysmal nocturnal haemoglobinuria; however, there are rare case reports of dysphagia secondary to intravascular haemolysis secondary to cardiac aetiology. There are currently two published case reports in the literature documenting the development of dysphagia secondary to intravascular haemolysis in patients with a left ventricular assistance device.^4,5^

This is a unique case for multiple reasons. Firstly, whilst intravascular haemolysis has been reported in the literature following TMVR with neo-LVOT obstruction, it is in the setting of significant paravalvular regurgitation. Our patient had only trivial paravalvular regurgitation, and it was postulated that the neo-LVOT obstruction was the most significant contributor to the development of intravascular haemolysis, similar to the pathophysiology responsible for LVOT obstruction in hypertrophic obstructive cardiomyopathy. Furthermore, dysphagia secondary to intravascular haemolysis has not been reported following TMVR. Whilst her presentation with dysphagia, neo-LVOT obstruction, and intravascular haemolysis was complex, the close relationship between these complications was successfully managed with simple fluid resuscitation, red blood cell transfusion, and up-titration of beta blocker therapy (*Summary figure*).

This case highlights several important complications following TMVR. Neo-LVOT obstruction is a relatively frequent complication of TMVR and whilst appropriate pre-procedural planning is an important factor in appropriate patient selection, haemodynamic factors are not always considered and may be difficult to predict. Whilst intravascular haemolysis following TMVR is rare, this case report highlights the complexity of managing patients with neo-LVOT obstruction with haemolysis and the complex interplay between certain haemodynamic factors. Dysphagia as a result of the intravascular haemolysis contributed to the vicious cycle of neo-LVOT obstruction and subsequent haemolysis.

## Lead author biography


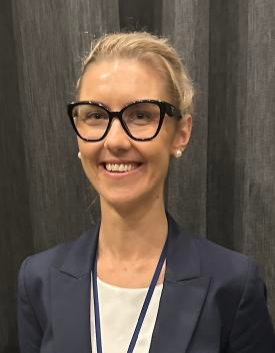


Dr Stephanie Sargent is a cardiologist currently undertaking a fellowship in echocardiography at The Prince Charles Hospital in Queensland, Australia. She completed an undergraduate degree in Biomedical Science at University of Sunshine Coast followed by the completion of Bachelor of Medicine/Surgery at James Cook University, Australia. She completed a Masters in Internal Medicine at the University of Sydney.

## Supplementary Material

ytaf081_Supplementary_Data

## Data Availability

No new data was generated or analysed in support of this research.

## Supplementary material

**Consent:** Written informed consent was obtained from the patient for publication of this case report and any accompanying images, in compliance with COPE guidelines.

**Funding:** None declared.
